# Comparative Genomics of *Histoplasma capsulatum* and Prediction of New Vaccines and Drug Targets

**DOI:** 10.3390/jof9020193

**Published:** 2023-02-02

**Authors:** Paula Cristina Silva Almeida, Bruno Stelmastchuk Roque, Andrei Giacchetto Felice, Arun Kumar Jaiswal, Sandeep Tiwari, Vasco Azevedo, Mario León Silva-Vergara, Siomar de Castro Soares, Kennio Ferreira-Paim, Fernanda Machado Fonseca

**Affiliations:** 1 Program of Multi-Professional Residence, Federal University of Triangulo Mineiro, Uberaba 38025-440, Brazil; 2 Post-Graduate Program in Tropical Medicine and Infectology, Federal University of Triangulo Mineiro, Uberaba 38025-440, Brazil; 3 Post-Graduate Program in Bioinformatics, Federal University of Minas Gerais, Belo Horizonte 31270-901, Brazil; 4 Post-Graduate Program in Microbiology, Institute of Biology, Federal University of Bahia, Salvador 40170-110, Brazil; 5 Post-Graduate Program in Immunology, Institute of Health Sciences, Federal University of Bahia, Salvador 40231-300, Brazil; 6 Department of Genetics, Ecology and Evolution, Federal University of Minas Gerais, Belo Horizonte 31270-901, Brazil; 7 Department of Infectious Diseases, Federal University of Triangulo Mineiro, Uberaba 38025-440, Brazil; 8 Department of Microbiology, Immunology and Parasitology, Federal University of Triangulo Mineiro, Uberaba 38015-05, Brazil; 9 Department of Biomedicine, Federal University of Triangulo Mineiro, Uberaba 38025-350, Brazil

**Keywords:** *Histoplasma capsulatum*, histoplasmosis, neglected fungal diseases, reverse vaccinology, drug target, comparative genomics, beta-1,3-glucanosyltransferase

## Abstract

*Histoplasma capsulatum* is a thermodymorphic fungus that causes histoplasmosis, a systemic mycosis that presents different clinical manifestations, ranging from self-limiting to acute lung infection, chronic lung infection and disseminated infection. Usually, it affects severely immunocompromised patients although immunocompetent patients can also be infected. Currently, there are no vaccines to prevent histoplasmosis and the available antifungal treatment presents moderate to high toxicity. Additionally, there are few options of antifungal drugs. Thus, the aim of this study was to predict possible protein targets for the construction of potential vaccine candidates and predict potential drug targets against *H. capsulatum*. Whole genome sequences from four previously published *H. capsulatum* strains were analyzed and submitted to different bioinformatic approaches such as reverse vaccinology and subtractive genomics. A total of four proteins were characterized as good protein candidates (vaccine antigens) for vaccine development, three of which are membrane-bound and one is secreted. In addition, it was possible to predict four cytoplasmic proteins which were classified as good protein candidates and, through molecular docking performed for each identified target, we found four natural compounds that showed favorable interactions with our target proteins. Our study can help in the development of potential vaccines and new drugs that can change the current scenario of the treatment and prevention of histoplasmosis.

## 1. Introduction

*Histoplasma capsulatum* is an environmental dimorphic fungus recovered from feces of bats, birds, and contaminated soil [1,2,3]. Histoplasmosis is a mycosis of worldwide distribution, with high prevalence in South American countries such as Brazil, Uruguay, Argentina, Ecuador, Venezuela and Paraguay. In North America, the disease is highly prevalent in the United States, in regions of the Mississippi and Ohio river valleys, although cases are still underdiagnosed, even in countries with high Gross Domestic Product (GDP) [4]. In Brazil, the number of people infected by *H. capsulatum* has been constantly increasing over the last few years, especially in immunocompromised patients [5,6,7].

Human infection occurs through the inhalation of microconidia present in the environment, which affect the pulmonary alveoli, where they acquire their yeast form at physiological temperature [8]. In the lung microenvironments, *H. capsulatum* is promptly phagocytosed by alveolar macrophages (CD11c+). In vitro studies have shown that in both human and murine macrophages, numerous yeast-like cells appeared by day 3 post-infection. However, transformation of conidia to yeasts was significantly restricted in human dendritic cells (DC) and murine lung DC which demonstrates their ability to significantly restrict the growth of yeast until 5 days post-infection [9,10]. This capacity of DC in restricting the infection is related to the considerable phagolysosomal fusion in DC that have phagocytosed viable yeasts, in contrast to the minimal amount of phagolysosomal fusion that occurs in human macrophages [11].

The acute immune response against infection also includes inhibiting the growth of the fungus by releasing the contents of neutrophils’ cytoplasmic granules [12]. Subsequently, phagocytosed yeasts are able to survive and proliferate as facultative intracellular pathogens due to their ability to inactivate reactive oxygen and nitrogen species released into the vacuole, prevent phagosome acidification and lysosomal fusion, and produce and transport needed nutrients to overcome nutritional immunity of the macrophage [13,14]. Therefore, the activation of infected mononuclear phagocytes by pro-inflammatory cytokines, such as IFN-γ, TNF-α and GM-CSF plays a pivotal role in the control of histoplasmosis. These cytokines induce the death of the phagocytosed yeasts cells, or stimulate the formation of granulomas which will trap the yeasts in the infected tissue [12]. Thus, yeasts of *H. capsulatum* are found in host tissues, with an optimal growth rate at 37 °C. This process can occur inside or outside the phagocytes [8,9].

Histoplasmosis can present several clinical manifestations, ranging from oligosymptomatic to acute or chronic lung infection, to disseminated infection. The development of clinical manifestations as well as their severity is determined by the immunological status of the host, the amount of inhaled inoculum, and the virulence of the strain [15,16,17,18]. The majority of the infected immunocompetent patients remain asymptomatic or develop only mild and nonspecific symptoms, which makes the diagnosis of histoplasmosis more difficult in these cases, where the disease is usually self-limiting [19]. On the other hand, in immunocompromised patients, clinical conditions can become progressively worse and result in a severe lung disease, or even disseminate to other organs such as liver, spleen, oropharynx, lymph nodes, skin, and adrenals [5,6,16,19]. In addition to the host immune status, the exposure to a variable amount of the inoculum can influence the progress of the disease [10]. Furthermore, there is also the possibility of developing a latency phase (chronic phase), in which *H. capsulatum* behaves as an opportunistic pathogen and can cause infection if the host presents a decrease in immunity [20,21,22,23].

Thus, considering the prevalence of histoplasmosis, its worldwide distribution, and the potential to develop serious or lethal disease, there is a clear need for studies aimed at the implementation of prevention, diagnosis and new strategies of treatment. Currently, options for treatment of histoplasmosis are based on drugs with moderate to high toxicity, especially when used for a long period. Treatment is indicated in cases with moderate or severe symptoms, or in immunocompromised patients [6]. The drugs of choice are itraconazole in moderate cases and liposomal amphotericin B, with the aim of reducing the renal toxicity in more complicated cases [23]. The duration of antifungal therapy varies according to the severity of the disease and the location of the fungus in the body, in many cases requiring the use of antifungal associations [24]. Thus, the search for new prevention and treatment options for histoplasmosis is important. More selective substances with specific action against fungal cells, could eventually decrease the toxicity of the current antifungal treatment.

There is a paucity of studies on reverse vaccinology, prediction of potential vaccines and drug targets on dimorphic fungi. Additionally, currently, there is a lack of vaccines for fungal diseases despite the attempts of some studies to look for targets of vaccines that may be effective in reducing the rates of systemic mycoses, among them, histoplasmosis [25,26,27,28]. The search for putative targets is a starting point for drug and vaccine development studies. In the case of a vaccine, this candidate also needs to be able to elicit a proper and accurate adaptive immune response. Thus, we aimed to identify some potential vaccine proteins and candidate drug targets against the pathogenic fungi *H. capsulatum* using comparative genomics, reverse vaccinology and molecular docking.

## 2. Materials and Methods

### 2.1. Identification of Data

The genome sequences of four *H. capsulatum* strains (Table 1) were retrieved from the GenBank database available at the National Center for Biotechnology Information (NCBI) (https://www.ncbi.nlm.nih.gov/genome/browse/#!/eukaryotes/243/, accessed on 5 January 2020).

### 2.2. Identification of Intra-Species Conserved Non-Host Homologous Proteins

The sequences obtained from NCBI were submitted to the OrthoFinder software, a commonly tool used in comparative genomics for developing accurate, comprehensive and fast analyses [29], which identified the regions of homology of the different strains of *H. capsulatum* and created orthogroups with the sequences of conserved proteins between the different strains. Subsequently, we performed subtractive genomics, where we first predicted the core genome, composed of conserved genes among the different strains used, and then compared the resulting dataset against human proteins. To avoid autoimmunity, the drug and/or vaccine candidate must be non-homologous to human proteins. An analysis was performed using BLASTall in which no “E value” was used. Thus, only proteins that did not have the slightest identity against the host were obtained in order to avoid autoimmunity responses or high toxicity of drugs to host cells.

### 2.3. Identification of the Subcellular Location of Proteins

The selected proteins were submitted to the software PSORT II Prediction to identify their subcellular location. This software uses the McGeoch method [30] to identify the location of a protein by predicting the presence of a signal peptide, formed by a central hydrophobic region and another charged N-terminal region. Then, a score is calculated from three determined values (length of the hydrophobic region, the peak value of this region and the net charge in the N-terminal charged region), and when this positive score is high, it indicates that there is a great possibility of having a signal sequence, cleaved or not. In this way, the selected proteins were classified as membrane protein, ribosomal, cytoplasmic, secreted, nuclear and from other locations (which included other organelles).

After the prediction of subcellular localization, the cytoplasmic proteins were analyzed by the MHOLline tool (http://www.mholline.lncc.br, accessed on 25 March 2020) and those classified as group G2 were used for docking, whereas the membrane and secreted proteins were analyzed in VaxiJen server, freely available in http://www.ddg-pharmfac.net/vaxijen/VaxiJen/VaxiJen.html, accessed on 14 February 2022.

### 2.4. Analysis of Protein Features

In addition to the information obtained from the NCBI database on the proteins of interest in the present study, the UniProt platform [31] was used to obtain functional information. Furthermore, the membrane proteins were submitted to an evaluation by the TMHMM software that determined, through a topology prediction by the Markov model, the number of transmembrane helices they possessed.

### 2.5. Identification of Vaccine Candidates

Membrane and secreted proteins were analyzed by VaxiJen 2.0, a tool that relies on the transformation of auto cross covariance (ACC) of protein sequences into uniform vectors, removing the influence of the length of the sequences. Immunogenicity and antigenicity were evaluated in order to determine their antigenic scores [30]. Proteins whose antigenic score for binding to the Histocompatibility Complex (MHC) molecules was greater than 0.51 were selected as possible vaccine candidates. The proteins were also analyzed in the Database of Essential Genes (DEG) [32] to analyze which proteins were essential for fungal cell survival, and only those that were predicted as essential were considered.

### 2.6. Identification of Drug Targets

The files containing all the amino acid sequences of the cytoplasmic proteins were submitted to MHOLline, an online tool that uses a set of programs to predict the structures of target proteins. Only those classified in the G2 group by MHOLline were included, thus using sequences with “high” and “good” quality. The structures grouped in the G2 group are those that present high levels of identity and were chosen for the molecular docking process.

Subtractive genomics was used to identify conserved targets essential to the fungus for drug target selection. The group of conserved core non-homologous to host and cytoplasmic proteins of *H. capsulatum* was submitted to the DEG, a database containing data from bacteria, archaea and eukaryotes that are composed of essential genomics currently reported for analysis of genes encoding proteins that are indispensable to cell life. The cutoff values used for BLASTp were: E-value = 0.0001, bit score = 100 and identity = 25% [32].

### 2.7. Comparison of Identified Vaccine Candidates and Drug Targets against the Newly Sequenced Genome of Histoplasma capsulatum and Available Transcriptomics Data

After performing all the subtractive genomics and reverse vaccinology approaches with genomes available at the beginning of the work (January 2020) we identified four vaccine candidates and four drug targets (see table in Section 3.3 and Section 3.4 respectively). We also performed the blast comparison (https://blast.ncbi.nlm.nih.gov/Blast.cgi, accessed on 12 January, 2023) of the identified vaccine candidates and drug targets against the newly published genome of *Histoplasma capsulatum* WU24 (GCA_017310585.1) to check if the identified vaccine candidates and drug targets were present in newly the sequenced strain WU24 (GCA_017310585.1). We performed BLAST analyses of the protein sequences against *H. capsulatum* genomes from FungiDB (https://fungidb.org/fungidb/app, accessed on 17 January 2023) to identify the coding genes and selected the transcriptomes of the strains H88, H143, G186AR, G186A and G217B derived from three different studies [33,34,35] to analyze the transcription of the genes.

### 2.8. File Preparation and Molecular Docking Analysis

After predicting protein structures from the MHOLline web server, the DoGSiteScorer web tool of Protein plus server [36] was used to identify active site binding pockets for each final protein target. Then, the MGLTools software suite, AutoDockTools (ADT) [37] was used to evaluate the best predicted 3D structures for structural errors, charges and after this process, a grid box was prepared in a specific region of the active site for the molecular docking process and saved in required pdbqt format for AutoDock Vina. After this process, a ligand library of 5008 natural compounds/products as druglike molecules, was downloaded [38] and prepared for molecular docking analysis according to Vilela Rodrigues, 2019 [39]. After the preparation, the software Autodock Vina command line was used for the docking analysis. Python script was used for the virtual screening process to identify top docked ligand with the protein targets [40].

Then, the Chimera visualization software [41] was used to analyze the interactions, identify the possible number of hydrogen bond interactions between protein targets and ligands, and extract the three-dimensional binding images.

## 3. Results

### 3.1. Identification of Intra-Species Conserved Non-Host Homologous Proteins

**Figure 1:**
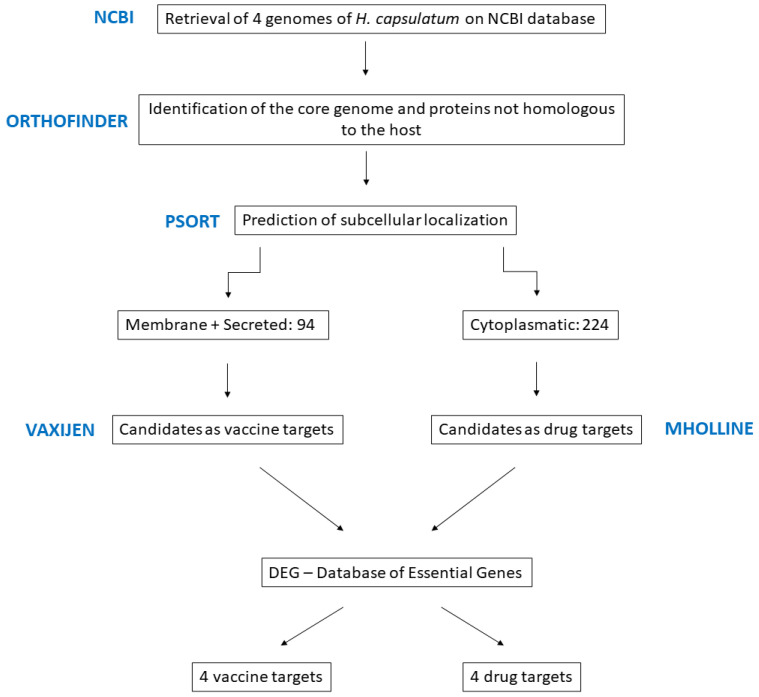
Workflow of methodologies used to select the drug and vaccine candidates. The bioinformatic tools and the total number of proteins are identified at each step.

All the steps performed are described in the workflow of Figure 1. The genomes of the four strains of *H. capsulatum* used here were compared, and the conserved genes among them were selected. These conserved genes were compared against the human host genome and we found 776 proteins shared by the four strains that were non-host homologous.

### 3.2. Prediction of Subcellular Location

The proteins were divided according to their subcellular location into: secreted, membrane, cytoplasmic, ribosomal and nuclear, totaling 776 proteins that did not have homology with human proteins (Table 2). The membrane (*n* = 49) and secreted (*n* = 45) proteins, due to their higher exposure to the host and more antigenic characteristics [42,43], therefore, had a greater capacity to activate the immune system, and were analyzed by VaxiJen software (http://www.ddg-pharmfac.net/vaxijen/VaxiJen/VaxiJen.html, accessed on 14 February 2022).

### 3.3. Prediction of Vaccine Candidates

For the analysis of vaccine candidates, of the ninety-four secreted and membrane proteins (*n* = 45 and *n* = 49, respectively), forty were analyzed for the probability of MHC I and II adhesion with a threshold of >0.51. Among them, only four were considered essential for fungal survival (DEG E-value < 10^−6^) when analyzed in DEG (Table 3). According to analyses on FungiDB data, except for EEH11056.1, which was not transcribed in the strains H88, H143 and G186AR, the other vaccine candidates were identified in all transcriptomics data available from strains H88, H143, G186AR, G186A and G217B [33,34,35]. Thus, these four proteins became our focus as good candidates for the development of vaccines, since their impairment may lead to the infeasibility of yeast survival in the human body. A total of three of the four essential proteins predicted to be good vaccine candidates were transmembrane, two of which had a transport function; EEH10718.1 a hypothetical protein encoded by the gene HCBG_00173 that transports oligopeptides, and EEH11056.1, a hypothetical protein encoded by the HCBG_00511 gene that also carries out transport through the cell membrane. The protein EEH09124.1 is a beta-1,3-glucanosyltransferase, that is coded by HCAG_05285 gene and could play an important role in the organization and structuring of the fungal cell wall, although this function has not been demonstrated. All the membrane proteins had their number of transmembrane helices described by THMM (Table 3). The only secreted protein that was identified as a good candidate for a vaccine target was EEH04925.1, encoded by the HCBG_06876 gene and involved in the cellular stress response process in transcription. All the vaccine target candidate proteins obtained in this study have a molecular weight lower than 100 kDa, which reinforces their high potential for being good candidates for vaccine development [44,45]. The information obtained from the proteins, such as name, gene and molecular weight were retrieved from UniProt [31].

### 3.4. Prediction of Drug Target Candidates

Of 776 non-host homologous proteins found, 224 were classified as cytoplasmic proteins. These were submitted to the MHOLline platform in order to predict the 3D structures of the proteins. Subsequently, only those that were grouped in the G2 group with “High” and “Good” quality classification were selected after the essentiality analysis in the DEG and considered as a drug target (Table 4). Concerning the FungiDB analyses, the candidate drug targets were all transcribed in strains H88, H143, G186AR, G186A and G217B [33,34,35].

### 3.5. Comparison of Identified Vaccine Candidates and Drug Targets against the Newly Sequenced Genome

The identified vaccine candidates and drug targets were compared by performing a blast search against *Histoplasma capsulatum* WU24 (GCA_017310585.1). We observed the blast comparison of drug targets EHH02668.1, EEH08858.1, EEH05968.1 and EEH04487.1 that showed 94% query coverage and 95% sequence identity (with protein QSS62328.1—hypothetical protein), 100% query coverage, 99% sequence identity (with protein QSS63647.1—chorismate synthase), 100% query coverage, 99% sequence identity (with protein QSS59260.1—imidazole glycerol phosphate synthase hisHF), and 100% query coverage, 96% sequence identity (with protein QSS62767.1—uricase), respectively. For vaccines we observed vaccine candidates EEH10718.1, EEH11056.1, EEH04925.1 and EEH09124.1 which showed 87% query coverage, 94% sequence identity (with protein QSS62142.1—hypothetical protein), 81% query coverage, 81% sequence identity (with protein QSS61807.1—MFS transporter, partial), 100% query coverage, 98% sequence identity (with protein QSS64714.1—stress response protein ish1, conidia-enriched transcript) and 100% query coverage, 96% sequence identity (with protein QSS63384.1 beta-1,3-glucanosyltransferase), respectively, in the newly sequenced genome of *H. capsulatum* WU24 (GCA_017310585.1).

### 3.6. The Docking Analysis Found the Possible Best Drug Targets

The four best drug targets found through subtractive genomics, along with the best natural compound druglike molecules were identified after the docking and virtual screening. Binding affinity, hydrogen bonds, and residues interactions are described in Table 5.

**Figure 2:**
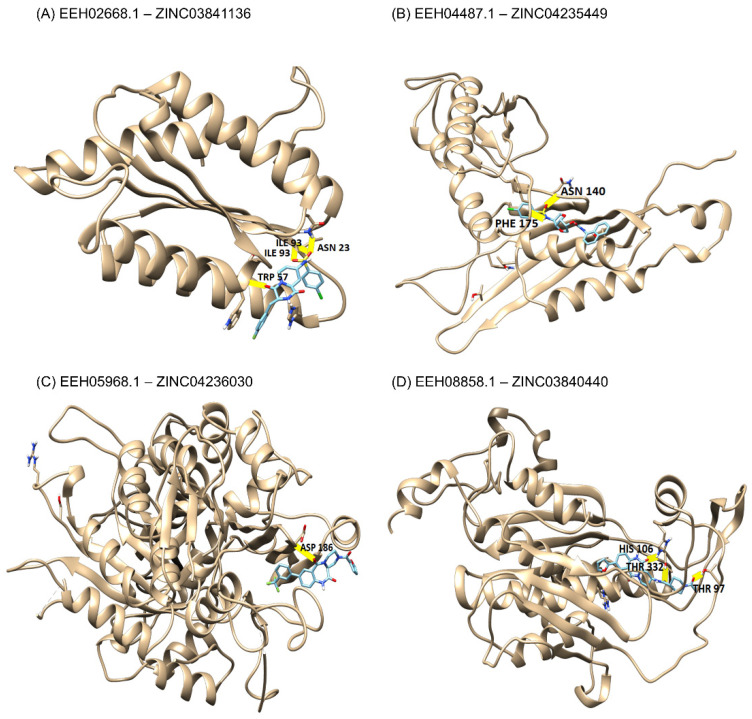
3D representation of each target’s interaction with its respective natural compound. In gold is represented the 3D structure of the drug target found. In shades of blue are represented the structures of natural compounds. Between the two structures are green stripes, which represented the hydrogen bonds of each interaction and next to each are described the residues of the active site that were part of this interaction.

Figure 2 represents the 3D images of drug targets and their interactions with natural compounds. The first target identified was 6,7-dimethyl-8-ribityllumazine synthase, represented by the locus EEH02668.1. Our analyses presented four hydrogen bonds, interacting with residues ASN 23, TRP 57, ILE 93, and a binding affinity with the compound ZINC03841136 of −10.1 (Figure 2, represented in A). The second identified target is represented by the EEH04487.1 locus, called uricase. In the molecular docking analyses, it interacted with residues ASN 140, PHE 175 with two hydrogen bonds, and presented a binding affinity of −10.0 with the compound ZINC04235449 (Figure 2, represented in B). The third target was imidazole glycerol phosphate synthase, represented by EEH05968.1, which had a binding affinity of −9.7 with the compound ZINC04236030 and interacted with one hydrogen bond with the residue ASP 186 (Figure 2, represented in C). Finally, the fourth target was chorismate synthase, represented by EEH08858.1. This target showed a binding affinity of −12.1 with the compound ZINC03840440, and three hydrogen bonds with the residues THR 97, THR 332, HIS 106 (Figure 2, represented in D).

## 4. Discussion

One of the most challenging aspects regarding invasive fungal infections is the increasing number of people living with immunosuppressed conditions such as HIV infection, solid organ transplants, and chronic use of corticosteroids. In those individuals, especially with AIDS, *H. capsulatum* usually causes life-threatening disseminated disease. In these patients, disseminated histoplasmosis progresses rapidly and is always fatal if untreated, with a lethality rate that can reach up to 50% [46]. For those cases, whether disseminated histoplasmosis occurs due to primary infection, reinfection, or dissemination of latent foci persisting after remote infection still remains unknown and controversial [23,47].

The human immune response against *H. capsulatum* can explain why immunosuppressed patients are so vulnerable. In healthy individuals, the recognition of the innate immune response by macrophages and dendritic cells is essential for the early production of cytokines, chemokines, and activation of phagocytosis in *H. capsulatum* infection. These innate cells carry out the function of effector cells and are required to promote Th1 cell differentiation and recruitment, while failure to generate this robust Th1 immune response leads to the collapse of immunity and fungi dissemination. The generation of Th2 or Treg cells is contrary to the development of protective immunity against the fungus and ends up neutralizing the action of Th1 cells. Clinical evidence supports the need for Th1 cells and TNF-α in host defenses against *H. capsulatum* [12,48].

Studies have shown that mice lacking T cells exhibited high mortality after a fungal infection with a small inoculum, proving the importance of CD4^+^ and CD8^+^ T cells in the effectiveness of the immune response against this pathogen [12,13]. The drop in CD4^+^ T cell counts during primary infection led to murine death, and the loss of CD8^+^ T cells decreased the efficiency of fungal elimination [49]. The opposite is seen after vaccination where CD8^+^ T cells conferred protection while CD4^+^ T cells become adjuvants in the response [50]. The elimination of both CD4^+^ and CD8^+^ T cells after 6 weeks of infection increases the fungal burden, showing the influence of these cells on reactivation [51]. The vaccination with recombinant Sec31protein also demonstrated a reduction in fungal burden and improved survival of mice infected with *H. capsulatum*, however, its effectiveness was critically dependent on the presence of Vb4^+^ T cells [52].

A pioneering study looking for proteins that could generate immunity against *H. capsulatum* was performed in 1991, where an extract of the cell wall and membrane in the yeast phase was prepared and used in murine models [53]. Subsequent research focused on analyzing specific fractions of the extract, and testing its antigenicity and immunogenicity [54,55]. A 62 kDa glycoprotein fraction isolated from the extract, HIS62, was identified as immunogenic. Additional experiments showed that approximately 80% of mice immunized with purified HIS62 survived the subsequent lethal intravenous inoculum of *H. capsulatum*. Those animals immunized with the native HIS62 antigen and then challenged with a sublethal inoculum of *H. capsulatum* may suffered from delayed hypersensitivity reaction (DTHR) [54]. Later, HIS62 became known as heat shock protein 60 (Hsp60) of *H. capsulatum* and its recombinant counterpart rHsp60 had an antigenicity and immunogenicity similar to the native extract, with 100% of the mice vaccinated with rHsp60 surviving a lethal intranasal inoculum with *H. capsulatum* yeasts [55,56]. Afterwards, it was shown that the effectiveness of immunization in mice with the Hsp60 protein was dependent on the production of IL-10 and IFN-γ [57].

In addition to the rHsp60 initial studies, different strategies have been used in order to develop an effective vaccine candidate against *H. capsulatum* (e.g., H antigen), a member of the β-glucosidase family, a surface protein of the fungi cell wall, showed no protection or limited protection when sublethal and lethal inoculum was used via intravenous or intranasal application, respectively [58,59]; priming of dendritic cells with apoptotic macrophages, which previously phagocytosed yeasts of heat-inactivated *H. capsulatum* followed by auto-transplantation of these dendritic cells to a murine model was able to produce protective CD4^+^ or CD8^+^ T cell responses in the murine model, but it was also invaluable when used in the context of HIV patients, who present with a lack of these cells [60]; an alkaline extract of the yeast phase of *Histoplasma* involved in glucan particle conferred protection dependent on IFN-γ and IL-17 and the neutralization of these cytokines weakened the efficacy of protection [61]. However, despite several attempts in developing an applicable vaccine against *H. capsulatum*, the results still need to be enhanced.

Here, a different approach was applied. Using the whole genome sequencing of four *H. capsulatum* strains (e.g., G186AR, H143, H88 and Tmu) and reverse vaccinology, we were able to select the commonly shared proteins that were not homologous to the human host. This strategy is an important issue that should be highlighted in a potential vaccine development scheme in order to increase the specificity of the immune response, minimizing the risk of autoimmunity reactions or even adverse effects. Using this bioinformatic approach, four proteins that were essential for the survival of *H. capsulatum* ranging from 510 to 759 amino acids were identified (three hypothetical and one beta-1,3-glucanosyltransferase). There were three (EEH1078.1, involved in oligopeptides transportation; EEH11056.1, involved in transport through the cell membrane; and EEH09124.1, the beta-1,3-glucanosyltransferase involved in the organization and structuring of the fungal cell wall) that presented transmembrane helices ranging from 1 to 14 and were specialized in cell membrane transportation. The remaining hypothetical protein (EEH04925.1) did not present transmembrane helices and was involved in the cellular stress response process in transcription. The locations of the three proteins exposed in the fungal cell membrane are promising, since these sites are usually the location where the recognition and host-pathogen interactions occur, thus establishing the infection. On the other hand, the proteins that were classified as cytoplasmic were referred for evaluation as possible targets of drug action, as they are generally associated with metabolic processes essential to cell survival. Similar to our results, recently, seven protein targets for putative vaccine design against *Candida auris* were described, including beta-1,3-glucanosyltransferase. The results also demonstrated that the vaccine candidates possess strong antigenic features [62].

One limitation of the applicability of two of the selected proteins (EEH1078.1 and EEH11056.1) is the presence of a high number of transmembrane helices, which could make the purification and isolation process on a large scale difficult but does not invalidate their potential as possible candidates [63]. In contrast, beta-1,3-glucanosyltransferase and the other secreted protein also meet all the established criteria as potential vaccine candidates. Secreted proteins can be involved in molecular interactions with host cells, enabling their survival, multiplication and dissemination, directly or indirectly. Membrane and secreted proteins perform essential functions for the survival of the microorganisms and have more organized amino acid regions, making the contact of these proteins with the antigen easier, and having greater potential to generate an effective immune response [42,43]. Some vesicular proteins, such as histone 2B and the heat shock protein Hsp60, have been shown to react with serum from patients with histoplasmosis, suggesting the involvement of these vesicles in host-pathogen interactions [64]. Proteomic analyzes of the contents of extracellular vesicles in *H. capsulatum* and *Cryptococcus neoformans* support the hypothesis that transport through vesicles is a general mechanism for transporting virulence-related macromolecules and plays an important role in host-pathogen interactions [65,66].

Several of the described virulence factors of *H. capsulatum* that have been identified in the secreted vesicles are unconventional cell wall components. For example, the surface M antigen is a catalase involved in the protection of fungal cells from oxidative stress [67]. Additionally, for *H. capsulatum*, vesicular bodies were observed in association with the cell wall and in the extracellular medium, suggesting the active use of vesicular transport for secretory processes. Therefore, the secretion of fungal extracellular vesicles is an important mechanism in fungal biology [64].

Reverse vaccinology has been used as a promising strategy for increasing the chances of finding an applicable target for vaccines against infectious diseases. The majority of the studies have been performed using bacterial genomes such as *Mycoplasma pneumoniae* [68], *Shigella dysenteriae* [69] and *Staphylococcus aureus* [70], but it has also been applied for viruses and fungi, such as Ebola [71] and *Candida tropicalis* [72], respectively. This bioinformatics approach has not only been used in order to increase the probability of finding a potential vaccine candidate, but also for the search of targets for new drugs. Discovery of new drugs in medical mycology is a crucial step towards decreasing the mortality rate of patients with systemic mycosis, especially due to the need for long periods of treatment (from weeks to months), usually with toxic drugs such as azoles and polyenes [23,72,73]. It should be noted that the development of a potential vaccine must be able to protect both immunocompetent and immunocompromised individuals. In case protective immunity against reinfection develops due to natural infection, live vaccines have been successful in promoting efficient and durable immune defenses in humans [74].

The MHOLline platform allowed us to identify four good drug target candidates (e.g., 6.7-dimethyl-8-ribityllumazine synthase, uricase, imidazole glycerol phosphate synthase and chorismate synthase), all of them predicted cytoplasmic proteins that were recognized as essential and linked to metabolic pathways indispensable to *H. capsulatum* (Table 4 and Table 5). It should be noted that the riboflavin biosynthetic process (Table 4) is an essential process to *H. capsulatum* virulence, thus, this biosynthetic pathway can represent an attractive possibility as a druggable target for this fungus [75]. Using molecular docking, a technique performed in silico based on discovering drugs by identifying new therapeutic compounds predicting ligand–target interactions at the molecular level [76], it was also possible to identify the high affinity interaction of these proteins (Table 5 and Figure 2) with their respective natural compounds (e.g., ZINC03841136, ZINC04235449, ZINC04236030 and ZINC03840440). Our results are promising, as these natural compounds could be used alone or even associated with the current available drugs in the treatment of histoplasmosis, although in vivo and in vitro studies are necessary to confirm their efficacy and toxicity in the human host.

## 5. Conclusions

In this study, we presented four candidate targets for the production of vaccines against the dimorphic fungus *H. capsulatum* using reverse vaccinology, a technique that has shown to be very promising and reliable in the development of vaccines for several other microorganisms. Beta-1,3-glucanosyltransferase, the enzyme involved in the elongation of the beta-(1-3)-glucans in several fungi seems to be the most promising candidate and should be tested in vivo in the near future in order to check their efficacy and safety as a vaccine against *H. capsulatum*. The molecular docking approach allowed the identification of four natural compounds which could virtually interact with essential proteins linked to metabolic pathways indispensable to *H. capsulatum,* and could be used as new drugs in the treatment of histoplasmosis. The expression of seven out of eight vaccine and drug target candidates in all five strains available on FungiDB, corroborates their classification as putative essential genes. However, more transcriptomics studies of strains isolated from different conditions and locations worldwide are still necessary. Thus, although we consider these proteins as potential vaccine candidates against *H. capsulatum*, new methodologies, in vivo and in vitro, should be employed as a way to validate the in silico findings.

## Figures and Tables

**Table 1:** General information about the four *Histoplasma capsulatum* strains used in this work.

Assembly ID	Strain	Size (Mb)	GC%	Protein ^#^	GenBank Number
ASM15011v1	G186AR	30.4	44.5	9.253	GCA_000150115.1
ASM15103v1	H143	38.9	41.5	9.547	GCA_000151035.1
ASM15100v2	H88	37.9	42	9.445	GCA_000115005.2
GCA_000313325.1	Tmu	46.1	39.4	*	GCA_000313325.1

* Not annotated in NCBI (GenBank). ^#^ The protein number refers to the number of proteins that were annotated in each of the genomes. This data is available from NCBI (GenBank).

**Table 2:** Subcellular localization of *Histoplasma capsulatum* proteins by PSORT.

Localization	Number of Proteins
Secreted protein	45
Membrane protein	49
Cytoplasmic protein	224
Ribosomal protein	114
Nuclear protein	344
Total	776

**Table 3:** Predicted vaccine candidates for *Histoplasma capsulatum*. VaxiJen predicted the number of transmembrane helices (TMHMM). The subcellular location was obtained from PSORT (MP-membrane protein; PS-secreted protein). The protein name, molecular weight (MW) and amino acids length (AA) were obtained from the Uniprot database.

Protein ID	Gene Product	Limiar VaxiJen (>0.51)	Localization	TMHMM	Lenght (AA)	MW (kDa)
EEH10718.1	Hypothetical protein	0.5642	MP	14	759	81.86
EEH11056.1	Hypothetical protein	0.5578	MP	10	450	50.14
EEH04925.1	Hypothetical protein	0.5507	SP	0	510	58.28
EEH09124.1	Beta-1,3-Glucanosyltransferase	0.5239	MP	1	527	57.39

**Table 4:** Predicted drug targets for *Histoplasma capsulatum*. Protein ID and name were obtained from NCBI Genbank database.

Protein ID	MHOLline G2 Group	Protein Name	Functions (MF-Molecular Function, BP-Biological Process)
EEH02668.1	High	6,7-dimethyl-8-ribityllumazine synthase	MF-6,7-dimethyl-8-ribityllumazine synthase activity. BP-riboflavin biosynthetic process.
EEH08858.1	High	Chorismate synthase	MF-Chorismate synthase activity. BP-Aromatic amino acid family biosynthetic process, Chorismate biosynthetic process
EEH05968.1	High	Imidazole glycerol phosphate synthase hisHF	MF-Glutaminase activity, Imidazole glycerol-phosphate synthase activity, Oxo-acid-lyase activity. BP-Glutamine metabolic process, Histidine biosynthetic process.
EEH04487.1	Good	Uricase	MF-Urate oxidase activity. BP-Purine nucleobase metabolic process, Urate catabolic process.

**Table 5:** Result of the molecular docking for each drug target identified with its respective compounds, binding affinity, hydrogen bonds, drug score and interaction with residues of the active site.

Locus Tag/Name	ZINC Id	Binding Affinity (Kcal/mol)	Hydrogen Bonds	Drug Score	Interactive Residues
EEH02668.1/6.7-dimethyl-8-ribityllumazine synthase	ZINC03841136	−10.1	4	0.68	ASN 23, TRP 57, ILE 93, ILE 93
EEH04487.1/Uricase	ZINC04235449	−10.0	2	0.81	ASN 140, PHE 175
EEH05968.1/Imidazole glycerol phosphate synthase	ZINC04236030	−9.7	1	0.8	ASP 186
EEH08858.1/Chorismate synthase	ZINC03840440	−12.1	3	0.8	THR 97, 332, HIS 106

## Data Availability

The genome sequencing data used were retrieved from GenBank database available at the National Center for Biotechnology Information (NCBI).
